# Cavernous sinus thrombosis with bilateral orbital vein involvement and diffuse ischemic retinopathy

**DOI:** 10.5935/0004-2749.20220077

**Published:** 2023

**Authors:** Diana H. Kim, Sana A. Bautista, Sonul Mehta, César A. Briceño

**Affiliations:** 1 Department of Ophthalmology, Scheie Eye Institute, University of Pennsylvania, Philadelphia, PA, USA

**Keywords:** Cavernous sinus thrombosis, Orbital diseases, Tomography, optical coherence, Humans, Case reports, Trombose do corpo cavernoso, Doenças orbitárias, Tomografia de coerência óptica, Humanos, Relato de casos

## Abstract

A 53-year-old man with a 3-day history of periorbital swelling and vision loss in the left eye was found to have septic cavernous sinus thrombosis with bilateral orbital vein involvement causing congestive orbitopathy. He was treated with an emergent canthotomy and cantholysis, intraocular pressure-lowering drops, antibiotics, anticoagulation, and serial examinations. Optical coherence tomography ultimately revealed diffuse ischemic destruction of both layers of the retina, which suggested occlusion of the ophthalmic artery or the short posterior ciliary arteries and central retinal artery without intracavernous internal carotid artery involvement. The patient remained without light perception in the left eye after treatment.

## Abstract (pt)

Um homem de 53 anos, com história de 3 dias de edema periorbital e perda de visão no olho esquerdo, apresentou trombose séptica do seio cavernoso com envolvimento bilateral das veias orbitais, causando uma orbitopatia congestiva. O paciente foi tratado com uma cantotomia e cantólise de emergência, colírios para redução da pressão intraocular, antibióticos, anticoagulantes e exames seriados. A tomografia de coerência óptica finalmente demonstrou destruição isquêmica difusa de ambas as camadas da retina, sugerindo uma oclusão da artéria oftálmica ou das artérias ciliares posteriores curtas e da artéria retiniana central, com ausência de envolvimento do segmento intracavernoso da artéria carótida interna. O paciente permaneceu sem percepção luminosa no olho esquerdo.

## INTRODUCTION

Septic cavernous sinus thrombosis (CST) is a rare, life-threatening process that can extend to an orbital vein^(1)^. Herein, we describe a rare presentation of septic CST involving thrombotic extension to the bilateral orbital veins that led to congestive orbitopathy. Optical coherence tomography (OCT) revealed diffuse ischemic retinal destruction, which suggested a novel mechanism of the permanent vision loss.

## CASE REPORT

A 53-year-old man with a 3-day history of left-sided periorbital swelling, left-sided vision loss, and confusion after incurring thermal facial burns was transferred from an outside hospital. In the outside hospital, computed tomography (CT) revealed periorbital edema related to orbital cellulitis and compartment syndrome warranting left lateral canthotomy.

On arrival, the patient was confused and presented with a visual acuity (VA) of 20/80 in the right eye and no light perception (NLP) in the left eye, an intraocular pressure (IOP) of 14 mmHg in the right eye and >50 mmHg in the left eye, an unreactive mid-dilated left pupil, an extraocular motility deficit in all gazes in the left eye, proptosis and ptosis with 2+ periorbital edema and erythema, and 3-4+ conjunctival hemorrhagic chemosis. Repeat CT revealed left posterior globe tenting, periorbital edema, extraocular muscle thickening, and bilateral sinus thickening. Emergent extension of lateral canthotomy and cantholysis of the left eye were performed, with a resultant reduction in IOP to 30 mmHg. Subsequently, maintenance IOP-lowering drops were started in the left eye. Intravenous (IV) administration of vancomycin and cefepime was initiated for gram-positive cocci bacteremia. Three days later, the right eye worsened, with an IOP of 21 mmHg, periorbital edema, and conjunctival chemosis, and conjunctival injection for which IOP-lowering drop therapy was started.

**Figure 1:**
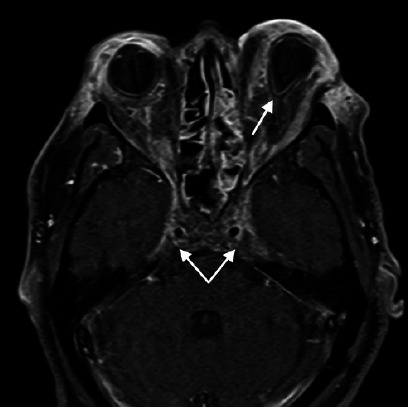
Axial T1-weighted magnetic resonance image of the orbits and head on day 5, demonstrating a bilateral cavernous sinus thrombosis (bottom arrows), as demonstrated by the lack of normal enhancement in the cavernous sinus and tenting of the left globe (top arrow), which is suggestive of increased orbital tension.

**Figure 2:**
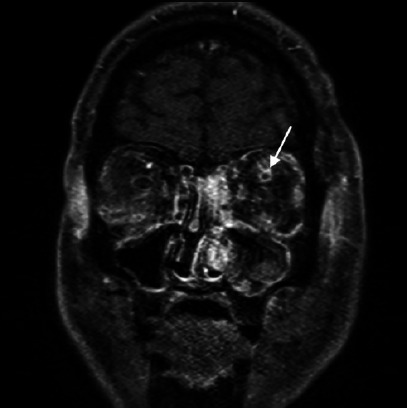
Coronal T1-weighted magnetic resonance image of the orbits and head on day 5, demonstrating a dilated left superior ophthalmic vein with an intraluminal non-enhancing thrombus (arrow), which is indicative of left superior ophthalmic vein thrombosis.

At five days, the patient agreed to undergo magnetic resonance imaging and venography (MRV), which revealed thromboses in the bilateral cavernous sinus (worse in the left eye), left superior ophthalmic vein, and right inferolateral orbital vein (Figures 1 and 2), for which heparin therapy was initiated. Blood cultures revealed methicillin-resistant *Staphylococcus aureus.* Therefore, cefepime was switched to ceftaroline for enhanced central nervous system penetration.

**Figure 3:**
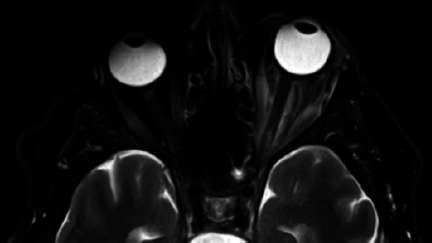
Axial T2-weighted magnetic resonance image of the orbits at 2 weeks, with improvement of the left superior ophthalmic vein thrombus and bilateral cavernous sinus thrombosis.

After 2 weeks, the patient’s VA was 20/40 in the right eye and NLP in the left eye, with improvement of clot burden on imaging (Figure 3). Heparin was transitioned to warfarin, and the IV antibiotics were continued for 6 weeks. At 6 weeks, the patient was discharged on warfarin.

**Figure 4:**
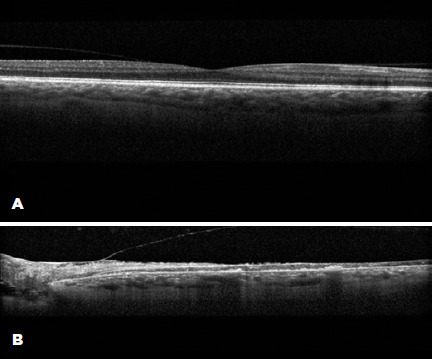
Optical coherence tomography image demonstrating an unremarkable macula of the right eye (A) and destruction of both the inner and outer retinal layers and hyperreflective bands within and below the retina in the left macula, presumably due to the profound ischemia resulting from the cavernous sinus thrombosis (B).

At 1-month follow-up, the patient was comfortable without eye pain. The examination revealed a VA of 20/20 in the right eye and NLP in the left eye, normal IOP, trace left lower lid ectropion and trace chemosis in the left eye, and notable pallor of left optic nerve, a fibrotic membrane extending across the macula, and sclerotic vessels. OCT revealed atrophy and destruction of both inner and outer layers of the retina in the left eye, consistent with sequelae of profound retinal ischemia (Figure 4). Fluorescein angiography was not performed. The patient was advised on monocular precautions and routine ophthalmic follow-up.

## DISCUSSION

Septic CST is a rare, potentially lethal disease caused by infectious thrombophlebitis, septic emboli, or sinusitis. While antimicrobials have greatly improved the management of septic CST, the associated mortality remains at 20%-30%, with serious ocular sequelae, including blindness in 8%-15% of cases^(2)^.

The cavernous sinuses are trabeculated dural spaces that drain the ophthalmic veins, middle and inferior cerebral veins, and sphenoparietal, and petrosal sinuses. They share an intimate anatomic relationship with cranial nerves III, IV, V1, V2, and VI, the sympathetic plexus, and the internal carotid artery. Compression of these structures and decreased venous drainage can therefore lead to unique findings, including external ophthalmoplegia from compression of CNIII, internal ophthalmoplegia due to loss of sympathetic and/or parasympathetic fibers from CNIII, and periocular/facial paresthesia and loss of corneal blink reflex^(3)^. Furthermore, decreased drainage from ophthalmic veins can lead to orbital compartment syndrome with periorbital edema, ptosis, proptosis, and chemosis, as was observed in the present case.

The progression to bilateral involvement in this case suggests the extension of the thrombosis from the left to the right via the intercavernous sinuses. While thrombosis of ipsilateral orbital veins has been shown in CST, bilateral involvement of the orbital veins is exceedingly rare, with only two reported cases of bilateral superior ophthalmic vein involvement in CST^(4,5)^. By contrast, the present patient had an orbital vein thrombosis that included the superior ophthalmic vein on one side and an inferolateral orbital vein on the other side. This highlights the bilateral asymmetrical extension of CST into the orbit, supporting retrograde extension as a result of increased intracavernous pressure even in the presence of ophthalmic venous valves^(6)^.

While the most frequently described etiology of vison loss in CST and resultant orbital compartment syndrome (OCS) is optic neuropathy^(7,8)^, a few cases with retinal ischemia and atrophy secondary to vascular occlusion have been reported^(9,10,11)^. This is the first case to demonstrate OCT findings of retinal ischemia and atrophy secondary to CST and OCS, confirming the notion that retinal arterial occlusion can in fact occur in OCS and CST and lead to visual decline, particularly in the setting of severely elevated IOP. Furthermore, OCT revealed a profound destruction of all retinal layers in the left eye in this case, implicating the involvement of either 1) both the central retinal artery (CRA) that supplies the inner retinal layers and short posterior ciliary arteries (SPCA) that supply the outer retinal layers or 2) the ophthalmic artery that supplies the two aforementioned arteries^(12)^. While occlusion of the intracavernous segment of the internal carotid artery (ICA) and/or CRA is known to cause visual impairment in CST^(13)^, occlusion of either the SPCA or ophthalmic artery without intracavernous ICA occlusion, as in the present case, has never been described, highlighting a unique pathophysiology of vision loss in CST.

Taken together, the findings of bilateral orbital vein involvement and diffuse retinal ischemia without intracavernous ICA occlusion add to the current understanding of the pathophysiology of CST and underscores the role of early recognition, prompt anticoagulation, IV antibiotic administration, and serial examinations.
